# Aggressive cutaneous vasculitis in a patient with chronic lymphatic leukemia following granulocyte colony stimulating factor injection: a case report

**DOI:** 10.1186/1752-1947-5-88

**Published:** 2011-03-01

**Authors:** Noha M El Husseiny, Mervat M Mattar

**Affiliations:** 1 Faculty of Medicine, Cairo University, Egypt

## Abstract

**Introduction:**

Vasculitis has been reported in a few cases of chronic lymphatic leukemia and with granulocytic colony-stimulating factor therapy. Those with granulocytic colony-stimulating factor occurred after prolonged therapy and there was a rise in total leukocyte count unlike that in our patient who received just a single injection for the first time.

**Case presentation:**

We report the case of a 64-year-old Egyptian man with chronic lymphatic leukemia who developed progressive cutaneous vasculitic lesions following injection of a single dose of a granulocytic colony stimulating factor before a third cycle of chemotherapy to improve neutropenia. This is an unusual case and the pathogenesis is not fully understood. Our patient was not on any medical treatment except for bisoprolol for ischemic heart disease. Although aggressive management with steroids, anticoagulation and plasmapheresis had been carried out, the condition was aggressive and the patient's consciousness deteriorated. A magnetic resonance imaging scan of his brain revealed multiple ischemic foci that could be attributed to vasculitis of the brain.

**Conclusion:**

The aim of this case report is to highlight the importance of monitoring patients on granulocytic colony-stimulating factor therapy, especially in the context of other conditions (such as a hematological malignancy) that may lead to an adverse outcome.

## Introduction

An adverse reaction has been reported in rare cases with granulocyte colony-stimulating factor treatment and in patients with hematological malignancies. Here we present a case of chronic lymphatic leukemia in which the patient received just one injection of lenograstin for neutropenia before starting the third cycle of chemotherapy, in the absence of other medical conditions. After the injection he developed cutaneous lesions and a skin biopsy revealed vasculitis. The condition was severe and the patient died 15 days after the onset of symptoms.

## Case presentation

**Figure 1:**
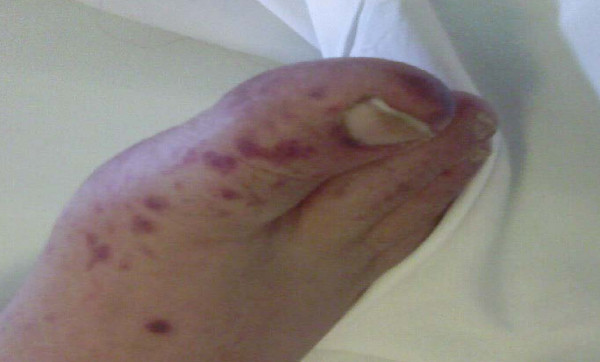
**Vasculitic lesions on the leg during the first day after lenograstin injection**.

**Figure 2:**
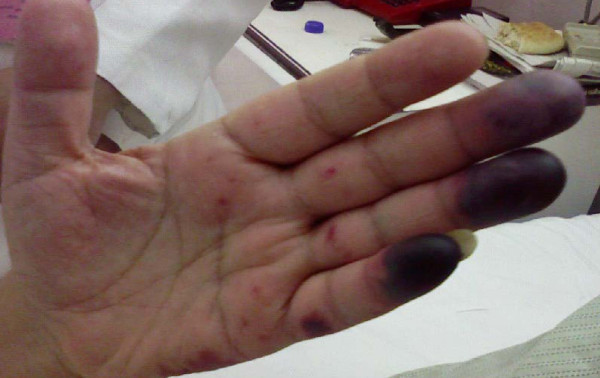
**Vasculitic lesions on the hand in the second day**.

**Figure 3:**
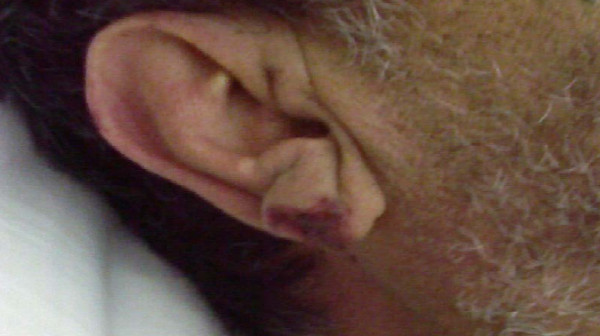
**Vasculitic lesions over the ear lobule**.

**Figure 4:**
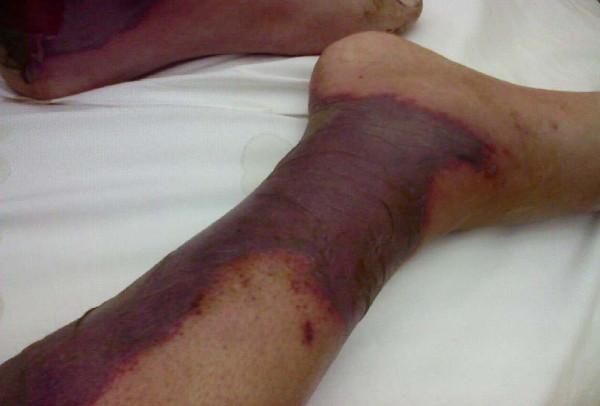
**Progression of vasulitic lesions on the lower limb after 4 days**.

**Figure 5:**
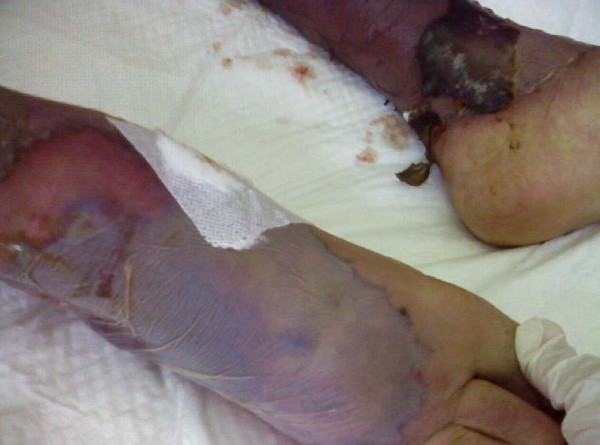
**The legs after 10 days of onset of treatment**.

**Figure 6:**
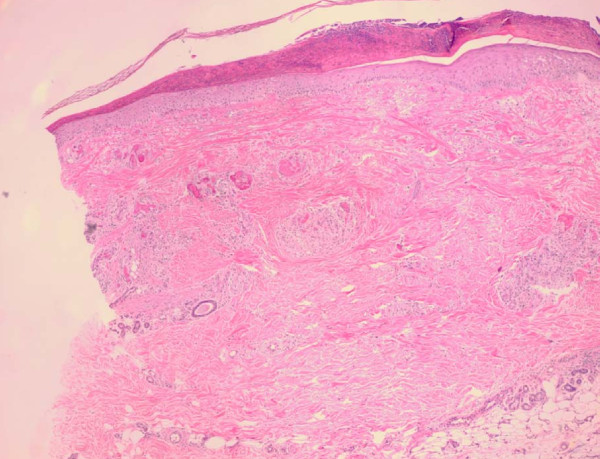
**Skin biopsy confluent necrosis in the epidermis**. Aggregates of lymphocytes perivascular. Intravascular fibrin clots.

A 64-year-old Egyptian man diagnosed with a case of B-cell chronic lymphatic leukemia (CLL); Stage III by RAI classification. He started a fludarabine and cyclophosphamide regimen for two cycles that passed smoothly. He was apparently healthy before the third cycle. Lenograstin was given before the third cycle as his TLC (total leukocyte count) was 2000/ul. After subcutaneous injection redness occurred over the tip of his nose, ears, hands and feet and within 48 hours lesions extended over his legs and arms (Figures 1, 2, 3, 4, 5]. Steroids and LMWH (low molecular weight heparin) were initiated; however, some red areas became blackish. Due to the aggressiveness of the condition daily plasmapheresis was performed, but without clinical improvement. The patient's level of consciousness deteriorated progressively until he passed into a deep coma and died five days after admission to the intensive care unit (15 days after the onset of the condition). Arterial and venous duplex were normal. A skin biopsy revealed confluent necrosis in the epidermis and infiltration of the dermis with lymphocytes around the blood vessels, which were occluded by fibrin plugs, a situation suggestive of vasculopathy (Figure 6). CBCs (complete blood count) revealed haemoglobin: 10 gm/dl, TLC 2000/ul(persistently) and platelet count 150,000/ul. The immune screen for cryoglobulins, cryofibrinogens, ANCA (antineutrophilic cytoplasmic antibodies), cold agglutinin, ANA (antineuclear antibodies), lupus anticoagulant and anticardiolipin were all negative. Tests revealed a PT (prothrombin time) of 19 seconds, PC (prothrombin concentration) of 56% INR (international normalized ratio) as 1.7 and a PTT (partial thromboplastin time) of 35 seconds. Fibrinogen repeatedly was normal. D (domain) dimer, carried out at 72 hours, was 4000 ng/ml. Protein electrophoresis showed hypoalbuminemia with increased β globulin. C3 was normal but C4 was consumed. No fragmented red blood cells (RBCs) were seen in blood film. CRP(C reactive protein) was 0.5 (n < 0.5), anti-HCV (anti hepatitis C virus antibodies) abs, HBs antigen and HBc antibodies were all negative. Serum viscosity was normal. Magnetic resonance imaging (MRI) of the patient's brain revealed age related brain involutional changes and a few tiny bilateral cerebral ischemic foci. Serum chemistry and electrolytes were normal apart from mild hyponatremia of 130 mEq/L. His blood culture was negative.

## Discussion

This case is considered unusual and the pathogenesis is not fully understood as the described vasculitis does not typically develop in this context with granulocytic colony-stimulating factor (G-CSF) treatment. Cutaneous vasculitis was reported in less than one in 7000 of those treated with G-CSF, and was moderate to severe in intensity. Most patients had severe chronic neutropenia and were on long-term G-CSF therapy. Symptoms developed with an increase in the absolute neutrophilic count (ANC) and stopped when the ANC decreased and the patients continued treatment at a reduced dosage [1]. Regarding the association of vasculitis with hematological malignancies, in a study done on 95 patients with hematological malignancy it was found that 22% had cutaneous vasculitis, 39% developed it concomitantly with malignancy, 26% of these before development of a malignancy and 35% after diagnosis of a malignancy. In 61% the vasculitis was explained only by the malignancy and in the remaining 39% it was explained by other factors, which included infection, cryoglobinemia and medications [2]. The association between lymphoma and vasculitis is rare in general. Lymphoproliferative disorders are the most common hematological malignancies associated with vasculitis (20%) especially hairy cell leukemia. Cases of vasculitis with CLL are rare (0.1 to 2%) [3]. The mechanisms of development of vasculitis in CLL include 1) immune complexes, 2) activation of B-lymphocytes, 3) antibodies directed toward endothelial antigens, 4) the direct effect of a malignancy on the vascular wall, 5) adverse reactions to anticancer drugs, and 6) CD5-positive B cells present in CLL may produce auto antibodies and monoclonal immunoglobulins with various autoantibody activities [4].

## Conclusion

In conclusion, patients should be monitored for development of inflammatory processes during G-CSF therapy, which should be given with caution especially in hematological malignancy situations.

## Consent

Written informed consent was obtained from the patient's next of kin for publication of this case report and accompanying images. A copy of the written consent is available for review by the Editor-in-Chief of this journal.

## Competing interests

The authors declare that they have no competing interests.

## Authors' contributions

NH participated in patient treatment and wrote the manuscript. MM was the chief supervisor on the patient's treatment and all decision-making in his treatment. MM also revised the manuscript for publication. All authors have read and approved the final manuscript.
